# Supplementary material for: Response of Prolyl 4 Hydroxylases, Arabinogalactan Proteins and Homogalacturonans in Four Olive Cultivars under Long-Term Salinity Stress in Relation to Physiological and Morphological Changes
Source: Cells. 2023 May 24;12(11):1466. doi: 10.3390/cells12111466 (PMC10252747; doi:10.3390/cells12111466)
Supplement: Supplementary file 1 [file cells-12-01466-s001.zip › cells-1953958-SI.pdf]

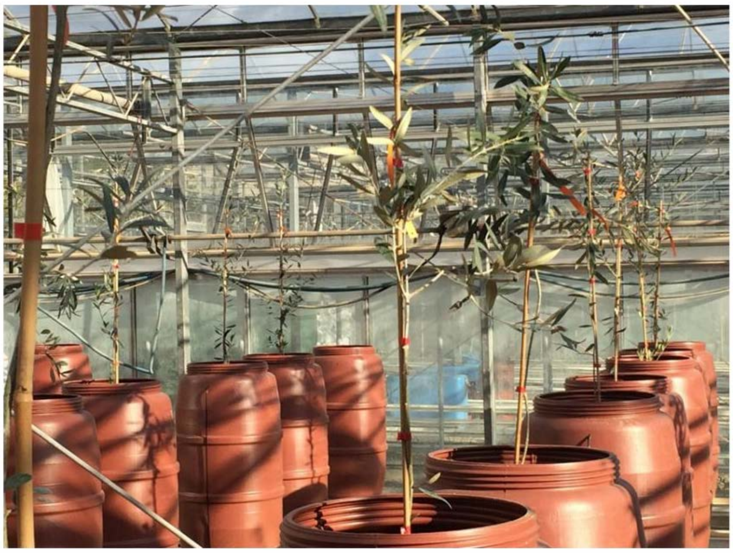

**Supplementary Figure S1.** Olive trees of Koroneiki, Lefkolia, Arvanitolia and Gaidourelia were placed in barrels of 90 cm long in four fertigation lines randomly for approximately one year to adapt to this environment and afterwards were subjected to NaCl treatment.

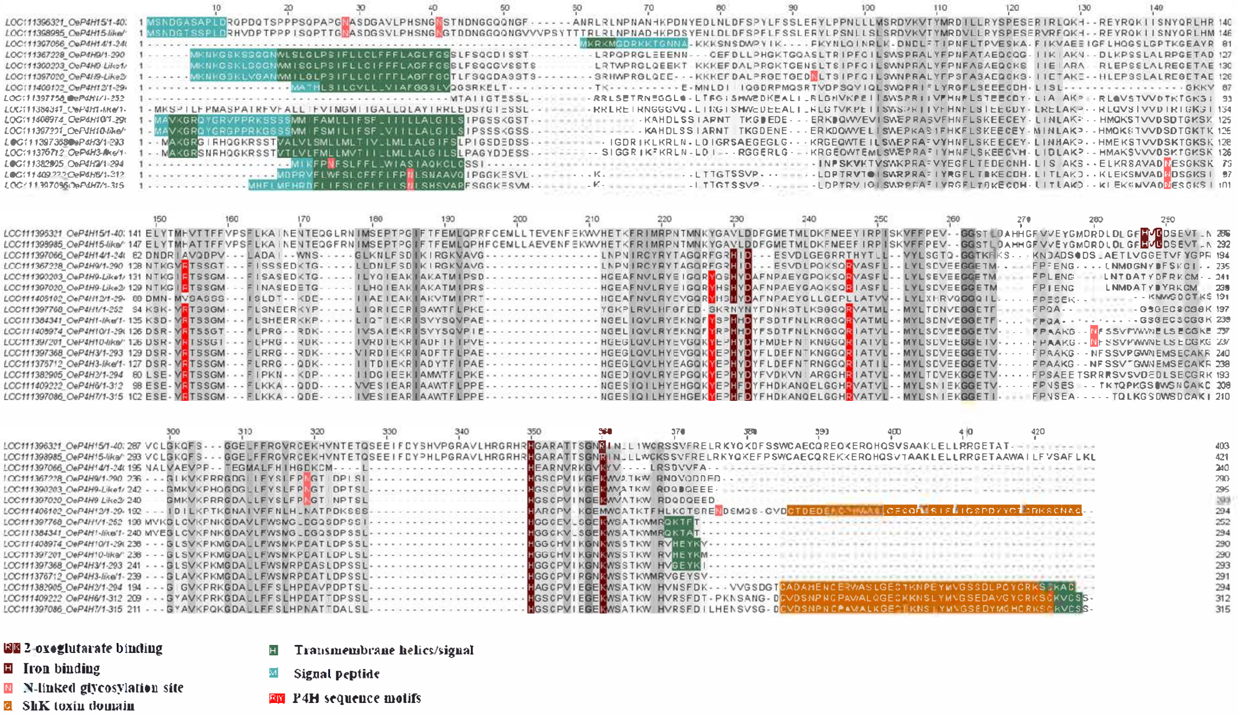


**Supplementary Figure S2.** CLUSTAL multiple sequence alignment of the *Olea Europea* putative P4Hs (OeP4Hs) amino acid residues highlighting the catalytic domain with its distinct functional domains and ER signal. The multiple sequence alignment shows the high similarity between P4H and P4H-like deduced amino acid sequences.

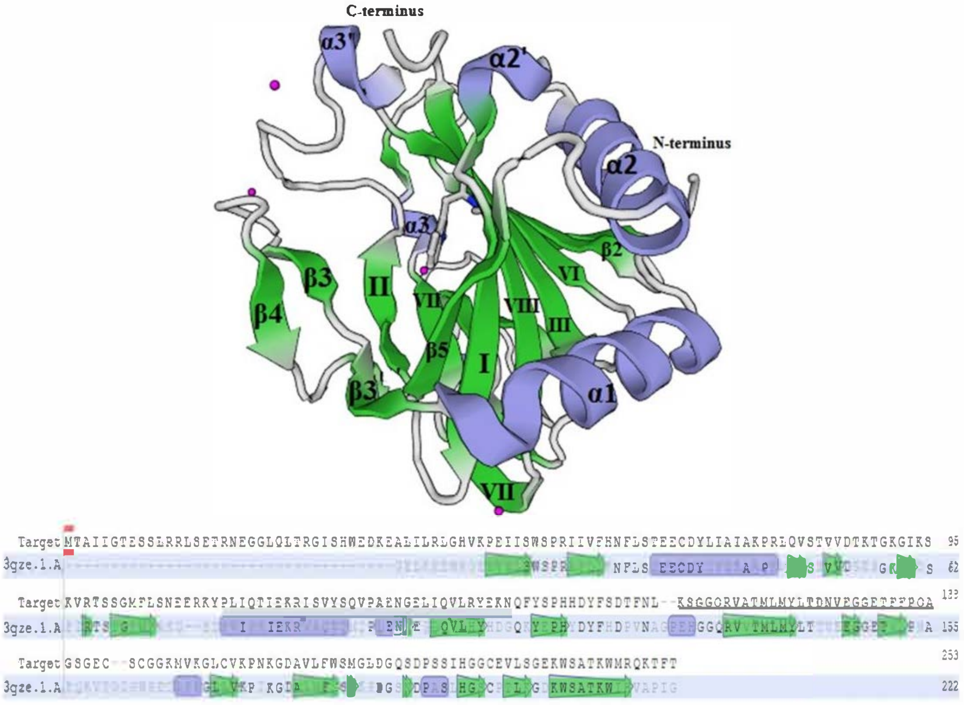


**Supplementary Figure S3.** Tertiary structure of OeP4H1 protein. The monomeric jelly-roll fold is indicated in green color with the numbers I-VIII. α helices and β strands are indicated on the sequence in purple and green, respectively.

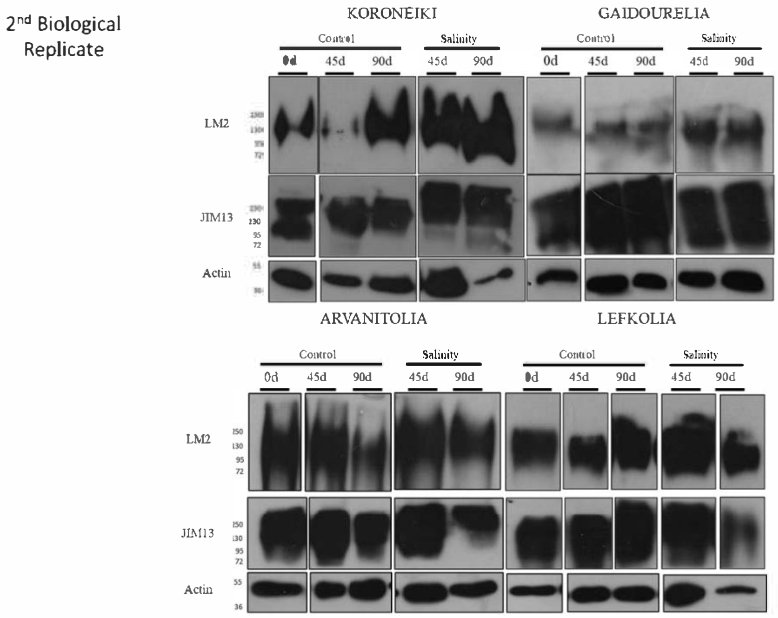


**Supplementary Figure S4.** Western blot analysis of LM2- and JIM13-bound AGPs in roots of Arvanitolia, Lefkolia, Koroneiki and Gaidourelia olive cultivars under a salinity time course of the second biological replicate. Total proteins were extracted from roots of four cultivars olive trees after 45 (45d) and 90 days (90d) of control (untreated) and salinity treatment and 15 μg from each sample were fractionated in SDS-PAGE for immunoblot analysis using LM2 and JIM13 antibodies and the actin antibody for loading control. The 0 day (0 d) refer to the un-treated control. The Molecular masses (KDa) are indicated on the left.

3rd Biological Replicate

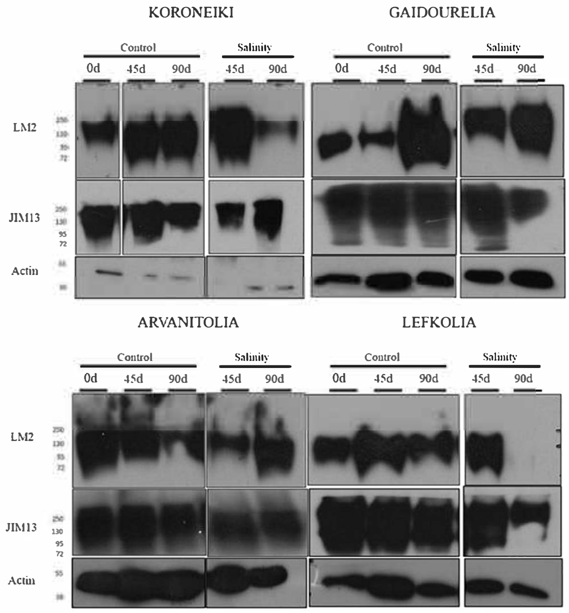


**Supplementary Figure S5.** Western blot analysis of LM2- and JIM13-bound AGPs in roots of Arvanitolia, Lefkolia, Koroneiki and Gaidourelia olive cultivars under a salinity time course of the third biological replicate. Total proteins were extracted from roots of four cultivars olive trees after 45 (45d) and 90 days (90d) of control (untreated) and salinity treatment and 15 μg from each sample were fractionated in SDS-PAGE for immunoblot analysis using LM2 and JIM13 antibodies and the actin antibody for loading control. The 0 day (0 d) refer to the un-treated control. The Molecular masses (KDa) are indicated on the left.

**Supplementary Table S1** Primary Protein sequence analysis of P4Hs and AGPs and protein domain prediction. P4Hc: Prolyl 4-hydroxylase, alpha subunit SM00702 (p4hc), ShKT: ShKT domain (IPR003582) SM00254 (ShkT_1), Fe2OG_Oxy: Oxoglutarate/iron-dependent dioxygenase (IPR005123) PF13640 (2OG-FeII_Oxy_3)

| *Olea europaea* | Gene ID | Chromosome | Protein length | Exons Number | Homology to Arabidopsis | P4Hc | Fe2OG_Oxy | ShKT |
|---|---|---|---|---|---|---|---|---|
| Oe_P4H1 | LOC111397768 | 7 | 253 aa | 11 | At_P4H1 | 54 - 248 | 132 - 249 | - |
| Oe_P4H1-like | LOC111384341 | un | 294 aa | 12 | At_P4H1 | 95 - 290 | 177 - 289 | - |
| Oe_P4H3 | LOC111397368 | 7 | 293 aa | 7 | At_P4H3 | 89 - 288 | 169 - 287 | - |
| Oe_P4H3-like | LOC111376712 | un | 291 aa | 7 | At_P4H3 | 87 - 285 | 167 - 285 | - |
| Oe_P4H2 | LOC111382905 | un | 294 aa | 7 | At_P4H2 | 40 - 241 | 120 - 240 | 284 - 294 |
| Oe_P4H6 | LOC111409222 | 14 | 312 aa | 8 | At_P4H6 | 58 - 256 | 139 - 255 | 302 - 312 |
| Oe_P4H7 | LOC111397086 | 7 | 315 aa | 8 | At_P4H7 | 62 - 258 | 143 - 257 | 305 - 315 |
| Oe_P4H9 | LOC111367228 | 18 | 293 aa | 8 | At_P4H9 | 88 - 286 | 172 - 285 | - |
| Oe_P4H9-like1 | LOC111390203 | un | 295 aa | 8 | At_P4H9 | 91 - 289 | 175 - 288 | - |
| Oe_P4H9-like2 | LOC111397020 | 7 | 293 aa | 8 | At_P4H9 | 89 - 287 | 173 - 286 | - |
| Oe_P4H10 | LOC111408974 | 14 | 290 aa | 7 | At_P4H10 | 86 - 285 | 166 - 284 | - |
| Oe_P4H10-like | LOC111397201 | 7 | 290 aa | 7 | At_P4H10 | 86 - 285 | 166 - 284 | - |
| Oe_P4H12 | LOC111406102 | 12 | 293 aa | 7 | At_P4H12 | 63 - 236 | 172 - 293 | 253 - 293 |
| Oe_P4H14 | LOC111397066 | 1 | 240 aa | 5 | At_P4H14 | 40 - 238 | 118 - 240 | - |
| Oe_P4H15 | LOC111396321 | 6 | 403 aa | 9 | At_P4H15 | 176 - 355 | 257 - 356 | - |
| Oe_P4H15-like | LOC111398985 | 8 | 421 aa | 9 | At_P4H15 | 182 - 361 | 263 - 362 | - |
| Oe_AGP4-like | LOC111390511 | un | 142 aa | 1 | - | - | - | - |
| Oe_AGP10-like | LOC111400351 | 10 | 182 aa | 3 | - | - | - | - |

**Supplementary Table S2** Protein sequence analysis and protein localization prediction * XXRR-like motif in the N-terminus; ** KKXX-like motif in the C-terminus; cytoplasmic domain: membrane topology, type 2 cytoplasmic tail.

| ***Olea europaea*** | **Gene ID** | **N-terminal signal peptide position** | **Cytoplasmic domain position** | **ER Membrane Retention Signals** | **Transmembrane helices position** | **N-Glycosylation position** |
|---|---|---|---|---|---|---|
| OeP4H1 | LOC111397768 | - | - | QKTF** | - | - |
| OeP4H1-like | LOC111384341 | - | - | QKTA** | - | - |
| OeP4H3 | LOC111397368 | - | 1 - 19 | AKGR*, GEYK** | 20 - 42 | - |
| OeP4H3-like | LOC111376712 | - | 1 - 19 | AKGR* | 20 - 42 | - |
| OeP4H2 | LOC111382905 | 1 - 19 | - | SCKA** | 4 - 23 | 6 , 73 |
| OeP4H6 | LOC111409222 | 1 - 23 | 1 - 5 | DPRV*, KVCS** | 5 - 23 | 19, 91 |
| OeP4H7 | LOC111397086 | 1 - 27 | - | KVCS** | 10 - 29 | 23, 95 |
| OeP4H9 | LOC111367228 | 1 - 35 | - | - | 13 - 35 | 258 |
| OeP4H9-like1 | LOC111390203 | 1 - 35 | 1 - 20 | - | 21 - 39 | 261 |
| OeP4H9-like2 | LOC111397020 | 1 - 35 | 1 - 11 | - | 12 - 34 | 76, 259 |
| OeP4H10 | LOC111408974 | 1 - 45 | 1 - 20 | VKGR*, HEYK** | 21 - 44 | 221 |
| OeP4H10-like | LOC111397201 | 1 - 45 | 1 - 20 | VKGR*, HEYK** | 21 - 44 | 221 |
| OeP4H12 | LOC111406102 | 1 - 24 | - | - | 5 - 24 | 244 |
| OeP4H14 | LOC111397066 | 1 - 15 | - | KRKM* | - | - |
| OeP4H15 | LOC111396321 | 1 - 40 | - | - | - | 28, 41 |
| OeP4H15-like | LOC111398985 | 1 - 40 | - | AFLK* | - | 28, 41 |

**Supplementary Table S3:** Table of primers used for the qPCR analysis.

| Primers for qPCR | Sequence |
|---|---|
| | |
| OeP4H1 F | 5’ GGGACTTTCTTTGCTAGGTG 3’ |
| OeP4H1 R | 5’ ACCGTTATTGTCGACCCATC 3’ |
| Oe P4H2 F | 5’ GCCTTCTGTAGACTCCGTTTG 3’ |
| OeP4H2 R | 5’ TCCCTGATCCCCATCAACTA 3’ |
| OeP4H3 F | 5’ CATGGAGGTTGCCCAGTTAT 3’ |
| OeP4H3 R | 5’ TCTGGAAAGCAGTTGACCAC 3’ |
| OeP4H6 F | 5’ CGGGAATTGACATCGTCGTA 3’ |
| OeP4H6 R | 5’ CCCCATGCCAAGTGTAATGA 3’ |
| OeP4H7 F | 5’ GGCCAATCAAGAACTAGGTGGTC 3’ |
| OeP4H7 R | 5’ TCACTGCCCTTAAGCTGAGTTG 3’ |
| OeP4H9 F | 5’ AGTGCAGGACGACGAAGATT 3’ |
| OeP4H9 R | 5’ CTTGTGAGAGATGACGGATG 3’ |
| OeP4H10 F | 5’ GGCTGCCATGTTATCAAAGG 3’ |
| OeP4H10 R | 5’ AGTGCTGCTGAAATCCTCGT 3’ |
| OeP4H1-like F | 5’ TTTTCTGGAGCATGGGGCTT 3’ |
| OeP4H1-like R | 5’ AGGTCGCAGTTTTCTGTCTC 3’ |
| OeP4H10-like F | 5’ AACAGAGGGTCAAGAATCGGT 3’ |
| OeP4H10-like R | 5’ TAACTTGATCTGCCGACGCA 3’ |
| OeP4H3-like F | 5’ AAGTTTGCATGGTGGTTGCC 3’ |
| OeP4H3-like R | 5’ ATTCCCCAGGCAGCTAAACC 3’ |
| OeP4H9-like F | 5’ TCTTCTCTCAGCAGGGAGTGT 3’ |
| OeP4H9-like R | 5’ CCGTGGCAAAGCATCAAATTCT 3’ |
| OeAGP4-like F | 5’ TTGCTGTGGCGTTGATGTAGTTT 3’ |
| OeAGP4-like R | 5’ GGGTGGAGAGAATAAAGACAAGGG 3’ |
| OeAGP9-like F | 5’ TACTCGGGCTCCAAGAAAGA 3’ |
| OeAGP9-like R | 5’ CTGATGCGGGACTAGGAGAA 3’ |
| OeAGP10-like F | 5’ CGCTCCAACACCATCTCCTT 3’ |
| OeAGP10-like R | 5’ AGGAGCAGGAGGTGAAGACA 3’ |
| Act7a F | 5’ AACGGAATCTCTCAGCTCCA 3’ |
| Act7a R | 5’ TTGCTTACGTGGCACTTGAC 3’ |

**Supplementary Table S4:** Table of antibodies used for immunolocalization of homogalacturonans and arabinogalactan proteins.

| Primary antibody | Epitope recognized | Secondary antibody |
|---|---|---|
| LM-20 | fully methyl-esterified HGs | FITC-conjugated anti-rat IgG |
| LM-19 | demethylesterified HGs and unesterified HGs. | FITC-conjugated anti-rat IgG |
| LM-18 | partially methyl-esterified HGs | FITC-conjugated anti-rat IgG |
| JIM-7 | partially methyl-esterified HGs | FITC-conjugated anti-rat IgG |
| JIM-5 | methyl-esterified and unesterified HGs | FITC-conjugated anti-rat IgG |
| JIM-13 | β -GlcpA-(1 → 3)-α -GalA-(1 → 2)-Rha | FITC-conjugated anti-rat IgG |
